# Investigating the correlation between neural and muscular activities during bilateral arm training in stroke survivors: A cross-sectional study

**DOI:** 10.4103/NRR.NRR-D-24-01279

**Published:** 2025-06-19

**Authors:** Yan Tang, Tara Scarlette Rosalyn Chen, Yi Xu, Pu Wang, Peng Dou, Dongfeng Huang

**Affiliations:** 1 Department of Rehabilitation Medicine, The Seventh Affiliated Hospital, Sun Yat-sen University, Shenzhen, Guangdong Province, China; 2 The First Affiliated Hospital, Sun Yat-sen University, Guangzhou, Guangdong Province, China; 3 Guangdong Engineering and Technology Research Center for Rehabilitation Medicine and Translation, Guangzhou, Guangdong Province, China

**Keywords:** bilateral arm training, coordination, entropy, functional connectivity, hemiparesis, interaction, near-infrared spectroscopy, neuroplasticity, stroke, surface electromyography

## Abstract

Stroke patients experience varying degrees of upper limb functional impairment. Although bilateral arm training can help stroke patients recover movement after stroke, little is known about the way in which the brain and muscles work together during this type of training. To address this, we conducted a cross-sectional study at The Seventh Affiliated Hospital, Sun Yat-sen University in China, where we observed the connection between brain and muscle activity during bilateral upper limb training in 21 stroke patients and 17 healthy controls. We used functional near-infrared spectroscopy and surface electromyography to measure changes in cerebral cortex oxygenation and upper limb muscle contraction signals, respectively. The results showed that, compared with the healthy control group, stroke patients had reduced functional connectivity and more irregular muscle activity in the affected flexor muscle during bilateral upper limb training. Moreover, we found a significant correlation between the surface electromyographic signal characteristics of upper limb muscles and cerebral oxygenation indicators of multiple brain regions in stroke patients. These findings indicate that bilateral upper limb training is an effective rehabilitation method that improves upper limb motor function in stroke patients by promoting brain functional connectivity and improving muscle activity patterns.

## Graphical abstract

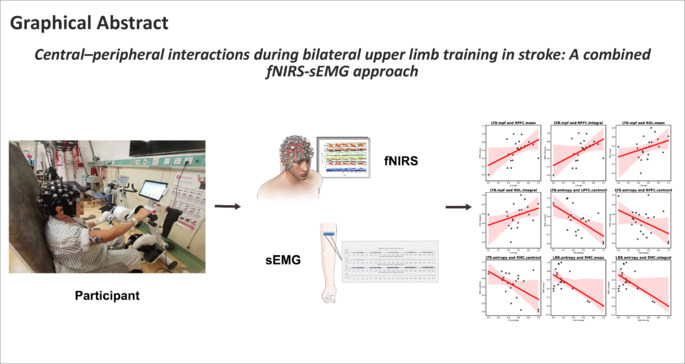

## Introduction

Stroke is a prevalent neurological disorder with high global incidence and disability rates (Wu et al., 2019). Approximately three-quarters of stroke survivors experience varying degrees of upper limb dysfunction (Dobkin, 2005), which profoundly impacts their quality of life and imposes a substantial societal burden (Ma et al., 2021). Post-stroke recovery of upper limb motor function is influenced by central and peripheral factors, including neuronal reorganization in both cerebral hemispheres and alterations in the coordination patterns between agonist and antagonist muscles in the upper limbs (Xi et al., 2021).

Bilateral arm training (BAT) has recently emerged as a widely implemented and extensively studied approach to stroke rehabilitation (Cauraugh et al., 2010; Chen et al., 2019). BAT has been empirically validated by clinical trials, which demonstrated that it led to significant improvements in post-stroke upper limb function (Cauraugh et al., 2010; Chen et al., 2019). Subsequently, researchers extensively investigated the central neural mechanisms underlying BAT, including the facilitation of contralateral cortical network reorganization (Luft et al., 2004) and the augmentation of interhemispheric functional connectivity within the motor cortex (Fan et al., 2015). These mechanisms collectively contribute to improved motor control of the affected limb and enhanced neuroplasticity in stroke patients (Chen et al., 2019). Parallel investigations into peripheral mechanisms have revealed attenuated coupling and reduced coordination between agonist and antagonist muscles in the affected upper limb of stroke patients (Luo et al., 2018; Yu et al., 2021). However, the interplay between muscle coupling dynamics and the central neural effects of BAT remains underexplored. Investigating the correlation between changes in electromyographic activity in agonist and antagonist muscles during BAT and concomitant brain activation could provide valuable insight regarding the influence of bilateral muscle activity on cortical function. Additionally, this line of inquiry could further clarify the central–peripheral interaction mechanisms of BAT, thus facilitating the development of more personalized rehabilitation strategies.

Functional near-infrared spectroscopy (fNIRS) is a non-invasive neuroimaging technique that measures neural activation according to relative changes in the concentrations of oxygenated hemoglobin (oxy-Hb) and deoxygenated hemoglobin (deoxy-Hb) on the cortical surface (Huo et al., 2024). Its portability, robustness against movement artifacts, and capacity for long-term monitoring of hemodynamic activity have led to its increasing use in assessing cerebral hemodynamics during stroke rehabilitation (Yang et al., 2019). Surface electromyography (sEMG), another non-invasive technique for monitoring muscle electrical activity, can also provide insight into key neural processes such as motor unit recruitment and synchronization in exercise rehabilitation (Al-Ayyad et al., 2023).

In this study, we performed fNIRS and sEMG to capture key signals of upper limb muscle contraction and cortical oxygenation. Our primary objective was to explore the correlation between cerebral oxygenation changes and electromyography signals during BAT according to the “central-peripheral-central” closed-loop theory of motor recovery (Jia, 2022). Our secondary objective was to compare differences in brain functional connectivity and upper limb EMG signals between stroke patients and healthy individuals. We hypothesized that we would find a linear correlation between upper limb electromyographic indices and cerebral oxygenation during BAT. We postulated that stroke patients would exhibit reduced brain functional connectivity, decreased sEMG amplitude, and increased sEMG signal complexity compared with healthy individuals. We anticipated that the results of this study would deepen our understanding of neuroplasticity in motor recovery post-stroke, and therefore inform the development of rehabilitation strategies.

## Methods

### Participants

In this cross-sectional study, we recruited 22 stroke patients from the inpatient unit at The Seventh Affiliated Hospital, Sun Yat-sen University, and 17 age-matched healthy controls without neurological diseases or arthralgia from the hospital cleaning staff. The participants were recruited between August 2023 and June 2024. One patient was excluded from the analysis because of excessive motion artifacts resulting from poor cooperation during the assessment. We ultimately analyzed data from 21 patients and 17 healthy controls. For the stroke patients, the inclusion criteria were as follows: (1) age 35 to 75 years; (2) stroke onset 2 weeks to 1 year prior to the study; (3) right-sided dominance according to the Edinburgh Handedness Inventory (Oldfield, 1971); (4) the presence of unilateral upper extremity paralysis according to clinical examination; (5) modified Ashworth scale (MAS) (Bohannon and Smith, 1987) less than grade II; and (6) sufficient cognitive function to complete the test. Participants who met any of the following criteria were excluded from the study: (1) the presence of severe comorbidities or concomitant diseases; (2) severe joint pain or joint deformities in the upper extremities; (3) unstable fractures of the upper limbs; and (4) a skull defect and/or damage to the skin covering the skull. The inclusion criteria for the control group were: (1) age 40 to 70 years; (2) no history of systemic diseases such as hypertension or diabetes mellitus based on standard diagnostic criteria; and (3) the absence of any upper limb joint injury or pain according to physical examination and medical history.

All subjects or their authorized relatives provided informed consent before study inclusion. The experimental procedure was approved by the Ethics Committee of the Seventh Affiliated Hospital, Sun Yat-sen University (Ethics approval No. 2019SYSUSH-019-01; dated November 7, 2022) and complied with the ethical standards specified by the *Helsinki Declaration* of 1975 (as revised in 2013). This study was registered in the Chinese Clinical Trial Registry (Registration No. ChiCTR-IOC-15006064).

### Surface electromyography preparation

First, according to the standard electrode sensor placement position recommended by Surface EMG for Non-Invasive Muscle Assessment (SENIAM) (Merletti and Hermens, 2000), we used a marker pen to locate and mark the locations of the bilateral long head of the biceps and the lateral head of the triceps on each participant. After cleaning the positioning area with an alcohol swab, Ag–AgCl bipolar electrodes were placed on the muscle belly of the target muscle with a constant distance of 20 mm between electrodes. We then recorded the sEMG signals using a BTS-FREEEMG1000 system (BTS Bioengineering, Milan, Italy) with a sampling rate of 1000 Hz per channel. The signals were band-pass filtered from 20 to 450 Hz.

### Functional near-infrared spectroscopy

**Figure 1:**
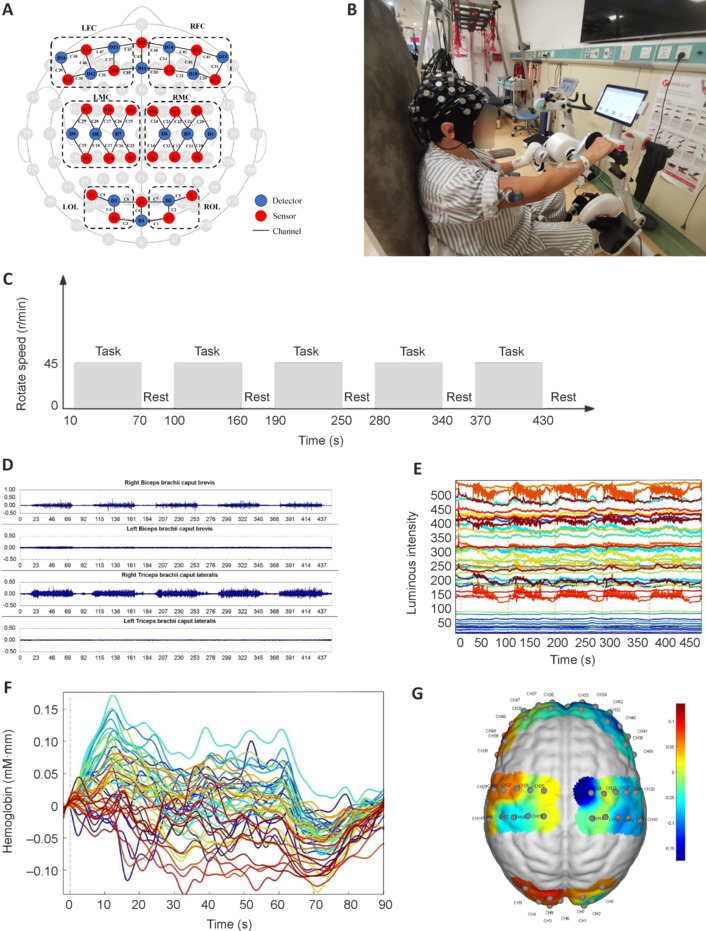
Experimental setting and block design flowchart. (A) fNIRS probe configuration (24 sensors, 16 detectors, 48 channels). (B) Bilateral arm training device. (C) Block design flow diagram. (D) Simultaneous sEMG signals of bilateral biceps and triceps. (E) fNIRS signal changes during training. (F) Block-averaged hemoglobin trend. (G) Three-dimensional brain map of mean oxyhemoglobin levels during training. fNIRS: Functional near-infrared spectroscopy; sEMG: Surface electromyography.

We used a multichannel fNIRS system (NirSmart-6000A, Danyang Huichuang Medical Equipment Co., Ltd., Danyang, China) to measure cerebral hemodynamic changes during the task. Signals were acquired with a sampling rate of 11 Hz, and the wavelengths were 730 and 850 nm. We positioned the 48 channels, including 24 sources and 16 detectors, over the bilateral frontal cortex, motor cortex, and occipital cortex according to the International 10–20 system. The fNIRS channels were classified into six regions of interest (ROIs) across both hemispheres: the left and right frontal cortex, the left and right motor cortex, and the left and right occipital cortex (**Figure 1A**). Each optode was fixed onto the scalp surface using a custom-made plastic cap, which was then covered with a black cloth to limit the influence of ambient light.

### Experimental protocol

Before the test began, the operator explained the entire experimental procedure to the participant, who was comfortably seated in front of the BAT device (**Figure 1B**). The BAT device used in this study primarily consists of a robust metal frame for structural support and stability, a motion drive system with pulleys and belts that enables the unaffected upper limb to drive synchronized and coordinated movements of the affected limb, and a screen-based control panel for real-time monitoring of movement speed and training duration to maintain a consistent exercise pace. For bilateral upper limb training, we utilized the interlimb coupling mechanism to promote repetitive, reciprocal (alternating) coordinated movements (Arya and Pandian, 2014; Wu et al., 2021). Interlimb coupling is a neural mechanism that integrates bilateral limb movements via structures such as the corpus callosum, reducing interhemispheric interference and enhancing neural connectivity. This mechanism promotes motor recovery and neuroplasticity in BAT by enabling the movement of the unaffected limb to drive synchronized and coordinated motion across multiple joints of the affected upper limb.

The chair and bilateral upper limb coordination device were adjusted so they were at a comfortable distance (**Figure 1B**) to ensure optimal comfort during exercise training. The participants were instructed to avoid significant head movements during the test and to monitor their speed, i.e., the “round/min” value on the screen, to maintain a steady pace of 45 rounds per minute. For stroke patients who were unable to grasp, an elastic band was wrapped around their wrists in a figure-eight pattern to secure their affected wrist to the handle of the BAT device. Before wrapping, their wrists were positioned in a neutral or light extension position to prevent injuries. The participants then rested quietly for 5 minutes before beginning the upper limb coordination training task. The entire process consisted of five 90-second modules, each with a 60-second task phase followed by a 30-second rest phase (**Figure 1C**). During the task, electromyogram signals from the bilateral biceps and triceps were collected in real time along with cerebral oxygenation data.

After completing the task, the stroke patients were evaluated using the relevant rehabilitation assessment scales, including the National Institute of Health stroke scale (NIHSS) (Adams et al., 1999) and the Fugl-Meyer Assessment of the Upper Extremity (FMA-UE) (Hernández et al., 2019), which is commonly used to assess the degree of upper limb motor dysfunction in clinical practice.

### Data analysis

#### Data preprocessing

We first filtered raw sEMG signals from the biceps brachii and triceps brachii (**Figure 1D**) using a fourth-order bandpass Butterworth filter (20–450 Hz) to remove motion artifacts, low-frequency baseline drift, and high-frequency noise. Power line interference and associated harmonics were subsequently filtered using a bandwidth-adjustable notch filter (50 Hz). The data underwent smoothing and normalization to enhance comparability. Because the acceleration at the initial stage of each exercise trial caused data instability, the initial 20-second period was eliminated and the following 40-second period was used for the final data analysis.

We analyzed the fNIRS data (**Figure 1E**) using the NirSpark software package (Danyang Huichuang Medical Equipment Co. Ltd.) after subjecting the data to the following four preprocessing steps. (1) Exclusion—if the test data contained severe artifacts that could not be removed by conventional methods, they were excluded; (2) Motion Correction—to correct motion artifacts, we implemented a moving standard deviation (SD) and spline interpolation technique that was repeated three times; (3) Filtering—to minimize noise, global trends, and biological signals such as respiration, cardiac activity, and low-frequency signal drift, we applied a bandpass filter with cut-off frequencies of 0.01–0.20 Hz based upon previous studies (Li et al., 2021); and (4) Conversion—we set all differential path-length factors to six and applied the modified Beer-Lambert law to convert optical densities into changes in oxy-Hb and deoxy-Hb concentrations. Because it was previously shown that oxy-Hb concentration displayed a superior signal-to-noise ratio relative to deoxy-Hb concentration (Kinder et al., 2022), we used oxy-Hb concentration for further analysis.

#### fNIRS data analysis

*Block average*

We calculated the trial block waveforms by setting the pre-baseline range to 0–10 seconds and the block range to 0–90 seconds (**Figure 1F**). To analyze the mean Oxy-Hb during the trials, we extracted 60-second time window from the active phase and compared them with 30-second rest periods to quantify its change between the task and baseline. This enabled us to calculate the change in Oxy-Hb between the task and baseline. We described the Oxy-Hb data in terms of the centroid, mean, difference, slope, and integral (the mean three-dimensional brain map is shown in **Figure 1G**).

*Functional connectivity analysis*

The left prefrontal cortex (channels 35–39 and 45–48), right prefrontal cortex (channels 30–34 and 40–44), left motor area (channels 15–19 and 25–29), right motor area (channels 10–14 and 20–24), left occipital lobe (channels 3, 4, 8, and 9), and right occipital lobe (channels 1, 2, 5, and 7) were selected as ROIs. For each resting-state dataset, the functional connectivity was analyzed by calculating the Pearson correlation between the time series of each channel-to-channel pair, resulting in a 48 × 48 matrix of R-values.

#### Surface electromyography data analysis

*Time-field analysis*

We used the integrated electromyography (IEMG) value and the root mean square (RMS) as time-domain features to elucidate the sEMG signals, which are indicative of muscle strength (Lawrence and De Luca, 1983). These were defined as


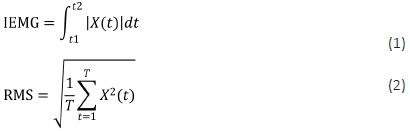


Where X(*t*) represents the myoelectric value at time *t*.

The IEMG encapsulates the cumulative electrical activity of a muscle over time, while the RMS indicates the amplitude of the EMG signal. Together, they provide a measure of the intensity and strength of muscle contractions. During motor tasks, increases in IEMG and RMS values may reflect the recruitment of more motor units or an increase in the synchronization of motor unit firing, which is associated with greater muscle strength.

We further analyzed the pre-processed data. A manual visual inspection was performed for each trial to determine the 40-second EMG signal segments with a consistent, steady flat response after RMS smoothing. We performed data computations using the Python programming language.

*Frequency-field analysis*

The median power frequency (MPF) is a usual parameter in the frequency domain of sEMG signals. It represents the frequency at which the power spectrum of the sEMG signal is divided into two halves, with half of the power occurring above and half below this frequency. Thus, it reflects the main frequency components and spectral structure of the time-series signal. The MPF is a crucial indicator in assessing muscle fatigue and changes in motor unit recruitment patterns (Svantesson et al., 1999). The calculation formula was as follows:


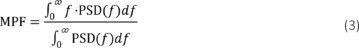


where PSD(*f*) represented the power spectral density.

As muscles undergo fatigue, there is a characteristic shift in the power spectrum towards lower frequencies (Svantesson et al., 1999). Therefore, monitoring the MPF can provide valuable information about the spectral composition of sEMG signals and aid in understanding the physiological changes that occur within the muscle during BAT tasks.

*Fuzzy entropy*

Fuzzy entropy (FE) serves as a complexity measure in the analysis of sEMG signals. By evaluating the degree of disorder within the signal, the FE quantifies the irregularity and unpredictability of a time series (Chen et al., 2007). Within the realm of sEMG, the FE captures the complexity of muscle activity patterns, indicating the system’s ability to adapt and react to diverse neuromuscular demands. As a new measurement, and compared with other measures of entropy, the FE has been shown to more effectively reflect sEMG signals (Simons et al., 2018).

We measured the FE based on a method proposed in a previous study (Chen et al., 2022), as follows:


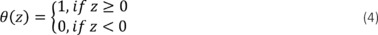


Given *N* data points from a time series {x(*n*)} = x(1), x(2),..., x(*N*), The FE can be calculated using the following algorithm:

For 1 ≤ *N* – *m* + 1, form m-vectors *X*_m_(i) were defined as:

X_m_(i)=[x(i),x(i+1),...,x(i+m–1)]–x_0_(i)      (5)

These vectors represent *m* consecutive *x* values, commencing with the point, with the baseline 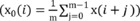 removed.

We defined the distance between vectors *X*_m_(i) and *X*_m_(j), *D*_ij, m_ as the maximum absolute difference between their scalar components.

Given *n* and *r*, we calculated the similarity degree *D*_ij, m_ of the vectors *X*_m_(i) and


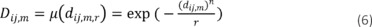


*X*_m_(j) with a fuzzy function;

We defined the function Φ_m_ as:


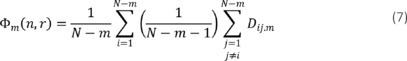


We increased the dimension to *m* + 1 to form vector *X*_m+1_(i), and subsequently obtained the function *f*_m+1_ by repeating steps 2–4.

For a time series with a finite number of samples *N*, the FE was estimated using the following equation:

FE(m,n,r,N)=lnΦ_m_(n,r)–lnΦ_m+1_(n,r)      (8)

### Statistical analysis

We statistically analyzed all experimental data using IBM SPSS Statistics for Windows, version 25.0 (IBM Corp., Armonk, NY, USA). The primary objective of this study was to investigate the correlation between cerebral oxygenation feature values and sEMG parameters. For this purpose, we adopted Pearson correlation analysis to evaluate the strength of the linear relationships between the variables, as in previous studies on brain activity coupling (Betzel et al., 2019).

The secondary objective was to compare the strength of the brain network functional connectivity between different groups at both the channel and ROI levels. The overall intergroup differences were evaluated using a repeated measures analysis of variance (ANOVA). To account for pairwise comparisons across multiple channels and ROIs, the *P*-values were adjusted using false discovery rate (FDR) correction to control the false discovery rate and family-wise error rate. Subsequently, the Tukey-Kramer test was performed to identify specific channels or ROIs with significant differences.

Another secondary aim was to compare between-group differences in sEMG values. We applied an ANOVA as above to identify significant variations in sEMG values, followed by a *post hoc* Bonferroni comparison test. The ANOVA evaluated overall group differences, while the Bonferroni test allowed for detailed comparisons between specific groups. A *P*-value of less than 0.05 was considered statistically significant for all statistical analyses.

## Results

A total of 21 stroke patients participated in this study, including 20 men and 1 woman. The clinical characteristics specific to the stroke patients are presented in **Table 1**. The healthy control group encompassed 17 age-matched adults and consisted of 7 men and 10 women. There were no statistically significant differences in age between the stroke group (53.0 ± 9.5 years) and the healthy controls (55.8 ± 3.9 years).

**Table 1:** Clinical characteristics of patients

No.	Age (yr)	Sex	Type of stroke	Course (mon)	Hemiparesis side	Site of lesion	FMA-UE	NIHSS	MAS
1	53	Male	Infarction	3	Left	Left cerebellum	65	4	0
2	53	Male	Hemorrhage	2	Left	Right thalamus and pedunculus cerebri	12	8	0
3	53	Male	Infarction	4	Right	Left basal ganglia	26	3	1
4	47	Male	Infarction	2	Right	Left basal ganglia	7	6	0
5	61	Male	Infarction	1	Right	Right pons	66	2	0
6	70	Male	Infarction	3	Right	Left basal ganglia	64	5	0
7	45	Male	Infarction	6	Left	Right frontal parietal temporal lobe	4	10	1
8	75	Male	Infarction	9	Left	Right basal ganglia	9	3	1
9	47	Male	Hemorrhage	12	Left	Right frontal parietal temporal lobe	17	2	1
10	56	Male	Infarction	4	Right	Left pons	56	2	0
11	60	Female	Infarction	0.5	Left	Right basal ganglia	16	8	0
12	55	Male	Infarction	0.5	Right	Left brainstem	56	4	0
13	36	Male	Hemorrhage	1	Left	Right parietal-basal ganglia	17	9	0
14	47	Male	Hemorrhage	1	Right	Parietal temporal lobe	12	9	0
15	49	Male	Hemorrhage	3	Left	Thalamus	6	1	1
16	45	Male	Infarction	0.5	Right	Callosum and right cerebellum	63	2	0
17	58	Male	Hemorrhage	3	Right	Left basal ganglia	46	9	1
18	50	Male	Infarction	3	Right	Left Frontal temporal lobe	61	3	1
19	65	Male	Infarction	1	Left	Right cerebral hemisphere	11	8	1
20	42	Male	Infarction	0.5	Right	Left frontal-periventricular	54	3	0
21	45	Male	Infarction	0.5	Right	Brain stem	66	1	0

FMA-UE: Fugl-Meyer Assessment of the Upper Extremity; MAS: Modified Ashworth scale; NIHSS: The National Institute of Health stroke scale.

### Functional connectivity and brain activation between stroke patients and healthy controls

**Figure 2:**
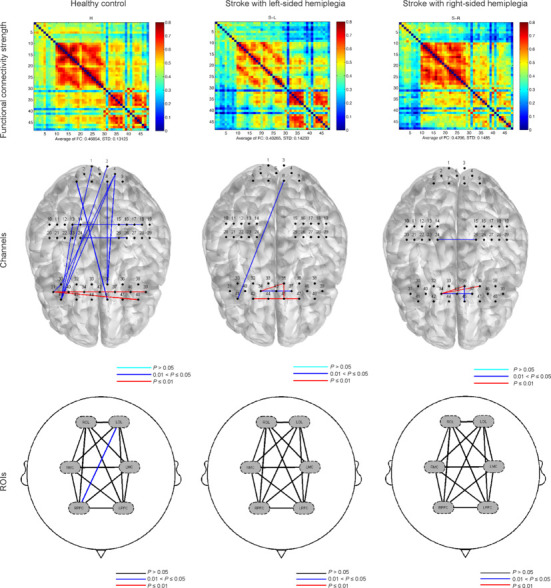
Functional connectivity heatmaps of the healthy control group and stroke groups with different hemiplegic sides, together with channels and ROIs. The average functional connectivity strength of the healthy control group was higher than that of the stroke group. In terms of channel and ROI levels, we found significant differences between the left hemiplegia group and the healthy control group. Blue lines represent channels with a *P*-value less than 0.05, while red lines indicate channels with a *P*-value less than 0.01. ROI: Regions of interest.

To determine whether there were differences in brain functional connectivity between the stroke patients and healthy controls, we analyzed the connectivity patterns at the channel level. Compared with the healthy controls, the patients with left hemiplegia exhibited weaker functional connections between channels, especially in the motor area **(Figure 2**). Interestingly, we also found that while the functional connections between motor channels in right hemiplegia patients were similar to those in healthy people, the spatial arrangement was not identical. However, functional connections between frontal regions in the right hemiplegic group were weaker than those in the other two groups (**Figure 2**).

To quantify these observations, we assessed the brain functional connectivity strength during bilateral upper limb motor tasks. The results showed that, relative to the healthy control group, patients with left hemiplegia exhibited a significant reduction (*P* < 0.05, FDR-corrected) in long-range functional connectivity strength between the right frontal lobe and left occipital lobe channels. In addition, as shown in **Figure 2** and **Table 2**, the reduction in connectivity strength between the bilateral frontal lobes was even more pronounced (*P* < 0.01, FDR-corrected). When comparing the right hemiplegic group to the control group, we observed fewer channels with statistically significant differences in connectivity strength (**Figure 2**). Nonetheless, as shown in **Table 3**, compared with the healthy controls, patients with right hemiplegia exhibited a statistically significant reduction (*P* < 0.05, FDR-corrected) in long-range functional connectivity strength between the right frontal lobe and left occipital lobe. Moreover, there was a more pronounced decrease in connectivity strength between the bilateral frontal lobes (*P* < 0.01, FDR-corrected).

**Table 2:** Channel pairs with significant differences in connectivity strength between healthy controls and stroke patients with left-sided hemiplegia

Ch-Ch	Connectivity strength		*F*	*P*-value (FDR corrected)
	Healthy control (*n* = 17)	Stroke with left-sided hemiplegia (*n* = 9)		
1–41	0.33±0.21	0.03±0.36	4.132	0.019
4–30	0.44±0.26	0.21±0.28	3.829	0.024
4–35	0.41±0.26	0.20±0.21	3.446	0.033
4–41	0.25±0.22	-0.002±0.3	4.316	0.016
5–45	0.46±0.24	0.23±0.25	3.051	0.048
8–35	0.46±0.22	0.20±0.27	4.571	0.014
13–18	0.71±0.15	0.54±0.22	3.011	0.049
24–26	0.69±0.17	0.42±0.39	3.983	0.032
30–31	0.55±0.17	0.32±0.33	3.265	0.04
31–39	0.63±0.20	0.34±0.25	6.153	0.003
31–48	0.65±0.20	0.34±0.27	6.277	0.004

Data are expressed as mean ± SD. Ch-Ch: Channel to channel; FDR corrected: false discovery rate-corrected.

**Table 3:** Channel pairs with significant differences in connectivity strength between healthy controls and stroke patients with right-sided hemiplegia

Ch-Ch	Connectivity strength		*F*	*P*-value (FDR corrected)
	Healthy control (*n* = 17)	Stroke with right-sided hemiplegia (*n* = 12)		
8–41	0.26±0.20	0.04±0.12	3.652	0.038
34–35	0.67±0.14	0.38±0.37	7.24	0.009
34–37	0.71±0.15	0.54±0.23	4.068	0.041
35–45	0.73±0.17	0.42±0.26	7.657	0.001
42–47	0.71±0.14	0.44±0.25	5.946	0.004

Data are expressed as mean ± SD. Ch-Ch: Channel to channel; FDR corrected: false discovery rate-corrected.

To further understand the effects on the hemiparetic side, we compared the brain functional connectivity between the left-sided and right-sided hemiparesis groups. We found significant differences in brain functional connectivity strength between the hemiparetic sides in stroke patients, as identified in **Figure 2** and **Table 4**. Specifically, compared with the group with right-sided hemiparesis, patients with left-sided hemiparesis had a statistically significant reduction (*P* < 0.05, FDR-corrected) in functional connectivity strength within channels 24 and 25. Similarly, there was a more pronounced reduction in connectivity strength between the bilateral frontal lobe channels (*P* < 0.01, FDR-corrected).

**Table 4:** Channel pairs with significant differences in connectivity strength between stroke patients with left- and right-sided hemiplegia

Ch-Ch	Connectivity strength		*F*	*P*-value (FDR corrected)
	Stroke with left-sided hemiplegia (*n* = 9)	Stroke with right-sided hemiplegia (*n* = 12)		
24–25	0.46±0.40	0.74±0.18	4.876	0.045
34–35	0.73±0.15	0.38±0.37	7.240	0.003
34–36	0.74±0.11	0.51±0.18	5.599	0.005
34–37	0.73±0.14	0.54±0.23	4.068	0.040
34–45	0.65±0.18	0.42±0.26	7.657	0.029

Data are expressed as mean ± SD. Ch-Ch: Channel to channel; FDR corrected: false discovery rate-corrected.

To further explore regional connectivity patterns, we examined statistical graphs of the ROIs. The results revealed that compared with the control group, the left hemiplegic patients showed a significant reduction in functional connectivity strength between the right frontal and left occipital regions (**Figure 2**). However, no significant differences were observed between the ROIs in the other two groups (**Figure 2**).

**Figure 3:**
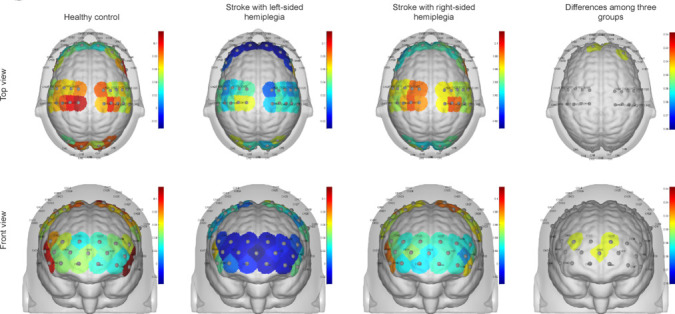
Cortical activation among healthy controls and stroke patients with different hemiplegic sides. Compared with the stroke group, the healthy control group had more symmetrical brain activation, followed by the right hemiplegia group.

To observe the differences in the activation levels of bilateral brain regions among the three groups during the BAT task, we performed a GLM analysis to obtain the β-value. The β-values of the bilateral brain regions in the healthy control group were relatively symmetrical, with significant activation observed. In contrast, the left hemiplegia group exhibited insufficient activation across the whole brain, with pronounced lateralization in motor areas (**Figure 3**).

These findings indicate that left-sided hemiparesis is associated with greater reductions in both functional connectivity and task-related activation, particularly within motor-related regions. This suggests a critical role of the hemiparetic side in shaping the neurophysiological patterns underlying stroke-induced motor impairments.

### Surface electromyographic changes between stroke patients and healthy controls

**Figure 4:**
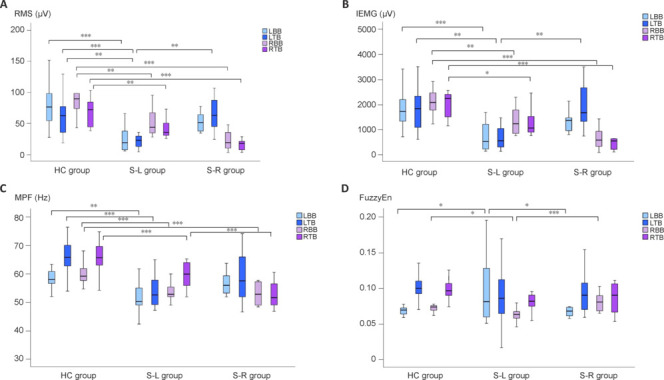
Difference in sEMG indices among healthy controls and stroke patients with different hemiplegic sides. Data are expressed as mean ± SD. *P < 0.05, **P < 0.01, ***P < 0.001 (one-way analysis of variance followed by Bonferroni correction). FuzzyEn: Fuzzy entropy; HC: healthy control; IEMG: integrated electromyography; LBB: left biceps brachii muscle; LTB: left triceps brachii muscle; MPF: median power frequency; RBB: right biceps brachii muscle; RMS: ROOT mean square; RTB: right triceps brachii muscle; S-L: stroke with left-sided hemiplegia; S-R: stroke with right-sided hemiplegia; sEMG: surface electromyography.

To assess the differences in muscle activity characteristics between stroke patients and healthy controls, we analyzed the RMS, IEMG, MPF, and FE values of the bilateral biceps brachii and triceps brachii muscles, as shown in **Figure 4** and **Table 5**. **Figure 4A** illustrates the RMS values of the bilateral biceps brachii and triceps brachii muscles in our three participant groups, providing essential information regarding the baseline muscle status of the study subjects. Consistent with expectations, the RMS values of the muscles on the affected side in left hemiparetic stroke patients were significantly lower than those in the control group. Similarly, the RMS values of the muscles on the affected side in right hemiparetic patients were significantly lower than those in the control group (*P* < 0.05). Furthermore, among the left hemiparetic patients, the RMS values of the left triceps brachii were notably lower compared with those in the right hemiparetic patients. Additionally, the RMS values of the healthy-side biceps brachii and triceps brachii in the left hemiparetic patients were markedly lower than those in the control group. Conversely, the muscles on the unaffected side in the right hemiparetic patients were not discernably different than those in the control group. These findings suggest that left hemiparetic stroke has a more pronounced impact on both the affected and unaffected-side muscle status compared with right hemiparesis.

**Table 5:** Surface electromyographic indexes of bilateral biceps brachii and triceps brachii among healthy controls and stroke with different hemiplegic sides

sEMG index		Healthy control (*n* = 17)	Stroke with left-sided hemiplegia (*n* = 9)	Stroke with right-sided hemiplegia (*n* = 12)
RMS (μV)	LBB	79.38±36.934	24.17±20.129	51.26±15.000
	LTB	65.41±35.400	20.76±10.799	64.66±27.433
	RBB	95.23±43.668	52.04±23.174	22.14±15.571
	RTB	76.74±42.069	45.32±24.473	20.39±20.330
IEMG (μV)	LBB	1928.58±839.531	737.26±564.940	1308.12±418.763
	LTB	1885.40±900.840	665.29±438.940	2080.36±1220.003
	RBB	2303.71±974.676	1357.47±562.442	642.90±429.464
	RTB	2211.34±972.075	1344.72±638.089	657.30±785.850
MPF (Hz)	LBB	58.56±4.517	52.71±6.931	56.57±4.089
	LTB	65.82±5.713	52.39±9.241	58.72±8.621
	RBB	60.13±4.401	53.54±3.143	52.88±4.014
	RTB	66.11±5.306	60.69±7.092	52.49±4.277
FE	LBB	0.068±0.009	0.098±0.049	0.067±0.006
	LTB	0.099±0.018	0.092±0.046	0.093±0.030
	RBB	0.073±0.008	0.063±0.009	0.080±0.012
	RTB	0.095±0.018	0.084±0.019	0.095±0.040

Data are expressed as mean ± SD, and were analyzed by Bonferroni correction. FE: Fuzzy entropy; IEMG: integrated electromyography; LBB: left biceps brachii muscle; LTB: left triceps brachii muscle; MPF: median power frequency; RBB: right biceps brachii muscle; RMS: root mean square; RTB: right triceps brachii muscle; sEMG: surface electromyography.

To further explore time-domain differences in muscle activation, we evaluated the IEMG values of the bilateral biceps brachii and triceps brachii muscles in the three groups, as depicted in **Figure 4B**. These values closely resembled those in **Figure 4A**. As anticipated, the IEMG values of the affected-side muscles in left hemiparetic patients were significantly lower than those in the control group (*P* < 0.05). A similar pattern was observed in right hemiparetic patients, where the IEMG values of the affected-side muscles were notably lower than those in the control group. Additionally, the IEMG values of the healthy-side muscles in the left hemiparetic patients were markedly lower than those in the control group, whereas no such differences were observed in the right hemiparetic patients. These findings indicate that left hemiparetic stroke exerts a greater influence on muscle activation, including on the contralateral side.

To investigate changes in muscle frequency characteristics, we analyzed the MPF values of bilateral muscles across the groups, as shown in **Figure 4C**. The results demonstrated that the left hemiplegia group exhibited lower MPF values in the affected biceps and triceps compared with both the healthy control group and the right hemiplegia group. Similarly, the right hemiplegia group showed lower MPF values in the affected muscles compared with the healthy control group. Moreover, patients with left hemiplegia displayed a significant decrease in the MPF of their healthy biceps, which highlighted a distinct asymmetry in the frequency characteristics.

Finally, to evaluate muscle complexity during the BAT tasks, we analyzed the FE values of the biceps and triceps muscles among the three groups (**Figure 4D**). In patients with left hemiplegia, the FE of the affected biceps was notably higher than that in the healthy subjects and right hemiplegia patients. Similarly, we found that in right hemiplegia patients, the FE of the biceps on the affected side was significantly greater than that in healthy subjects and left hemiplegia patients. Although no marked differences in triceps FE were observed among the three groups, the distribution of triceps FE values on the affected side showed greater variability, and this was also evident for the biceps on the affected side. These results indicate that both left and right hemiparetic stroke can lead to increased muscle fatigue in the affected biceps, with left hemiparesis triggering more pronounced changes.

### Correlation between neural and muscular activities in stroke patients

To explore the correlations between neural and muscular activities during BAT in stroke patients, we conducted a Pearson correlation analysis between cerebral oxygenation metrics and sEMG parameters (**Table 6**). The results indicated a high proportion of significant correlations between the MPF and entropy values of sEMG signals and the cerebral oxygenation metrics in various brain regions in stroke patients. Specifically, in the time-domain analysis of sEMG parameters, we observed significant correlations as follows: between the IEMG and RMS of the right triceps brachii and the metrics of the right motor cortex and left occipital lobe, between the left triceps brachii and the right occipital lobe, and between the right biceps brachii and the right occipital lobe.

**Table 6:** Pearson correlation analysis between the sEMG indices of bilateral biceps and triceps and the brain oxygen feature values of six ROIs in healthy controls and stroke with different hemiplegic sides

		LBB					LTB					RBB					RTB			
						
		IEMG	RMS	MPF	FE		IEMG	RMS	MPF	FE		IEMG	RMS	MPF	FE		IEMG	RMS	MPF	FE
LFC	Mean	–0.198	–0.051	0.255	–0.102		–0.02	0.005	0.318	–0.139		–0.042	–0.017	0.015	0.052		–0.152	–0.172	–0.146	–0.224
	Difference	0.091	0.082	0.087	0.256		–0.098	–0.095	0.262	0.261		0.031	0.056	0.083	0.275		0.067	–0.004	–0.268	0.226
	Slope	–0.225	–0.087	0.141	–0.386		–0.247	–0.235	0.24	–0.303		0.26	0.299	0.386	–0.026		0.003	–0.023	0.247	–0.078
	Integral	–0.198	–0.051	0.255	–0.102		–0.02	0.005	0.318	–0.139		–0.042	–0.017	0.015	0.052		–0.152	–0.172	–0.145	–0.224
	Centroid	–0.075	0.049	0.175	–0.376		0.061	0.115	–0.15	–0.585*		0.044	0.013	–0.142	–0.236		0.034	0.104	0.229	–0.249
RFC	Mean	0.209	0.341	0.294	–0.331		0.047	0.127	0.434*	–0.162		0.149	0.177	0.24	0.102		0.052	0.077	0.053	–0.231
	Difference	0.279	0.286	0.132	–0.01		–0.047	–0.02	0.27	0.101		0.222	0.265	0.461*	0.193		0.313	0.246	–0.032	0.233
	Slope	0.059	0.116	0.075	–0.311		–0.201	–0.123	0.396	–0.215		0.315	0.343	0.288	0.006		0.159	0.158	0.364	–0.096
	Integral	0.209	0.341	0.294	–0.331		0.046	0.127	0.436*	–0.162		0.149	0.177	0.246	0.102		0.052	0.077	0.057	–0.231
	Centroid	–0.094	–0.026	–0.205	–0.22		0.143	0.146	–0.264	–0.436*		0.037	–0.006	–0.205	–0.113		–0.151	–0.149	–0.139	–0.292
LMC	Mean	0.024	0.114	0.326	–0.302		0.19	0.216	0.257	–0.107		–0.157	–0.165	0.164	0.261		–0.22	–0.234	–0.24	–0.146
	Difference	0.142	0.219	0.111	–0.32		0.308	0.335	0.086	–0.059		–0.24	–0.252	0.301	0.498*		–0.222	–0.27	–0.403	0.16
	Slope	–0.032	0.006	0.125	–0.397		–0.109	–0.101	0.121	–0.206		0.133	0.133	0.334	0.158		–0.056	–0.058	0.098	0.038
	Integral	0.024	0.113	0.326	–0.302		0.19	0.216	0.257	–0.107		–0.157	–0.165	0.164	0.261		–0.22	–0.234	–0.24	–0.146
	Centroid	0.169	0.365	0.381	–0.37		0.224	0.256	0.446*	–0.138		0.083	0.142	–0.077	0.068		–0.175	–0.107	0.047	–0.176
RMC	Mean	0.169	0.231	0.271	–0.661*		0.284	0.303	0.273	–0.216		–0.142	–0.126	0.221	0.231		–0.347	–0.279	–0.146	–0.136
	Difference	0.053	0.221	0.275	–0.700*		0.282	0.325	0.179	–0.107		–0.251	–0.244	0.374	0.458*		–0.538*	–0.525*	–0.259	0.135
	Slope	0.096	0.15	0.149	–0.613*		0.127	0.132	0.189	–0.196		0.035	0.055	0.258	0.223		–0.239	–0.177	0.036	–0.002
	Integral	0.169	0.231	0.271	–0.661*		0.284	0.303	0.273	–0.216		–0.142	–0.126	0.221	0.231		–0.347	–0.279	–0.146	–0.136
	Centroid	0.204	0.33	0.262	–0.549*		0.299	0.345	–0.023	–0.523*		–0.144	–0.175	–0.058	0.111		–0.182	–0.186	0.024	–0.21
LOL	Mean	–0.307	–0.148	0.187	–0.143		–0.092	–0.076	0.234	–0.055		–0.168	–0.117	–0.092	0.104		–0.403	–0.387	0.004	–0.028
	Difference	0.091	0.185	0.1	–0.287		0.366	0.326	0.082	0.094		–0.22	–0.213	0.005	0.362		–0.515*	–0.460*	–0.16	0.042
	Slope	–0.281	–0.211	0.009	–0.069		–0.243	–0.275	0.084	0.029		0.231	0.264	0.149	0.082		–0.221	–0.177	0.281	–0.041
	Integral	–0.307	–0.148	0.187	–0.143		–0.092	–0.076	0.234	–0.055		–0.168	–0.117	-0.092	0.104		–0.403	–0.387	0.004	–0.028
	Centroid	–0.282	–0.157	–0.016	–0.204		–0.362	–0.264	0.037	–0.306		0.087	0.128	0.115	–0.171		0.077	0.097	0.354	–0.122
ROL	Mean	–0.278	–0.099	0.153	–0.620*		–0.087	–0.017	0.475*	–0.416		0.086	0.128	0.105	0.031		–0.363	–0.34	0.265	–0.172
	Difference	0.087	0.114	–0.165	–0.500*		0.038	–0.016	0.022	–0.229		0.348	0.351	0.235	–0.098		–0.197	–0.132	0.199	–0.073
	Slope	–0.203	–0.158	–0.253	–0.297		–0.463*	–0.449*	0.139	–0.254		0.465*	0.483*	0.161	–0.111		–0.119	–0.105	0.462*	–0.113
	Integral	–0.278	–0.099	0.153	–0.620*		–0.087	–0.017	0.475*	–0.416		0.086	0.128	0.105	0.031		–0.363	–0.34	0.265	–0.172
	centroid	–0.249	–0.09	0.287	–0.118		0.156	0.163	–0.044	–0.174		–0.195	–0.179	0.073	0.054		–0.029	–0.022	0.01	–0.147

* Indicates a significant difference in values, P < 0.05. FE: Fuzzy entropy; IEMG: integrated electromyography; LBB: left biceps brachii muscle; LFC: left frontal cortex; LMC: left motor cortex; LOL: left occipital lobe; LTB: left triceps brachii muscle; MPF: median power frequency; RBB: right biceps brachii muscle; RFC: right frontal cortex; RMC: right motor cortex; RMS: root mean square; ROI: regions of interest; ROL: right occipital lobe; RTB: right triceps brachii muscle; sEMG: surface electromyography.

In the frequency-domain analysis of sEMG parameters, significant correlations were found between the MPF of the left triceps brachii and the metrics of the right frontal lobe, left motor cortex, and right occipital lobe.

Furthermore, based on the FE analysis, we identified substantial correlations between the FE values of the left biceps brachii and the metrics of the right motor cortex and right occipital lobe, between the FE values of the left triceps brachii and the metrics of the right motor area and bilateral frontal lobes, and between the FE values of the right biceps brachii and the metrics of the bilateral motor areas (**Table 6**).

These findings highlight the complex interplay between sEMG signal characteristics and cerebral oxygenation metrics in stroke patients and provide valuable insights into the neuromuscular and cerebral dynamics underlying stroke pathology.

## Discussion

Although researchers have in recent years increasingly focused on the central and peripheral effects of bilateral upper limb training, the mechanisms driving these effects have not yet been fully clarified (Renner et al., 2020; Chen et al., 2022). This study aimed to explore the relationship between upper limb electromyographic indices and cerebral oxygenation metrics during BAT in stroke patients. Our findings confirmed that there was a linear correlation between brain and muscle variables during BAT. In addition, our data supported our hypothesis that stroke patients would show a significant reduction in brain functional connectivity compared with healthy controls. Finally, we found that the affected extremity in stroke patients exhibited decreased IEMG and RMS, decreased MPF, and increased FE values in the flexor muscles. These observations offer a deeper insight into the central–peripheral mechanisms underlying BAT.

### Brain functional connectivity during bilateral arm training

The left hemiplegic patients showed reduced global functional connectivity, especially in the right frontal and left occipital regions during the BAT task. This aligns with prior work linking diminished connectivity to impaired motor recovery (Zhang et al., 2024). While the role of the right hemisphere in visuospatial processing (DiNuzzo et al., 2022) and the association of left hemiplegia with visuospatial deficits (Esposito et al., 2021; Overman et al., 2024) are known, our results highlight the impact of left hemisphere lesions on global connectivity during bilateral movement. This decline may reflect disrupted visual-motor integration, which could be worsened by subclinical visuospatial impairments (Bartolomeo, 2021). Lower FMA scores in left hemiparetic patients, along with the correlation between diminished interhemispheric connectivity and FMA scores (Tanamachi et al., 2023), strengthen this link. Monitoring brain functional connectivity during BAT could enable the objective evaluation of rehabilitation outcomes and predict recovery. Combining this with other markers like electroencephalography and longitudinal monitoring could lead to the further refinement of assessments and reveal connectivity changes linked to recovery and neuroplasticity.

### Surface electromyogram activities during bilateral arm training

Hemiplegic individuals exhibited reduced strength, particularly on the affected side, with left hemiplegic patients showing greater contralateral weakness. This aligns with prior work on bilateral muscle mass reduction and altered neural drive post-stroke (Nozoe et al., 2024). However, our findings highlight a novel asymmetry: greater contralateral weakness in left hemiplegia, potentially reflecting the left hemisphere’s dominant role in bilateral motor control and the impact of interhemispheric disruption (Merrick et al., 2022). From a rehabilitation perspective, these findings suggest that targeting bilateral motor pathways, rather than focusing solely on the affected side, may enhance recovery outcomes.

Left hemiplegic patients uniquely exhibited bilateral bicep fatigue, in contrast to the absence of fatigue in the unaffected limb of right hemiparetic patients. While reduced neuromuscular efficiency post-stroke has been established (Ma et al., 2017), and poorer motor performance in left hemiplegia has been documented (McCombe Waller and Whitall, 2005), our novel finding highlights the crucial role of lesion laterality in bilateral fatigue patterns. This asymmetry likely reflects the left hemisphere’s dominance in bilateral motor control (Zhou et al., 2021), where damage disrupts interhemispheric communication. Therefore, lesion laterality differentially impacts motor performance and fatigue, emphasizing the need to consider the unaffected side in individualized bilateral training protocols for optimal rehabilitation.

Furthermore, the hemiplegic patients showed increased bicep activity variability and complexity, possibly reflecting compensation. While the unilateral upper limb study reported lower flexor FE compared with extensor FE (Chen et al., 2007), and reduced FE in stroke patients overall (Ao et al., 2015), we unexpectedly found increased flexor FE on the hemiplegic side. This paradox likely arises from our focus on bilateral coordination, unlike prior work (Chen et al., 2007; Ao et al., 2015). Bilateral tasks involve complex interhemispheric interaction; synergistic movements (Zhao et al., 2023) can increase complexity and entropy. Meanwhile, passive flexor engagement during bilateral coordination (Hong and Newell, 2008) may also increase FE. These factors complicate the interpretation of muscle activity during bilateral stroke tasks.

### Correlation between neural-muscular activities

Our finding of a positive correlation between left triceps brachii fatigue and right frontal cortical activation during BAT offers novel insight into the interplay between peripheral and central fatigue in stroke. While MPF decline is a known correlate of muscle fatigue (Wang et al., 2023), our observation of increased brain activation during fatigue, particularly in the right frontal region, potentially reflects adaptive neuroplasticity and compensatory cortical reorganization (Cotter et al., 2021) in the context of BAT. This right frontal hyperactivation is consistent with fMRI findings of increased prefrontal activity during post-stroke fatigue (Duan et al., 2012), and, given the established fNIRS-fMRI correspondence (Jolly et al., 2023), supports our fNIRS findings. The positive correlation between left triceps MPF and right frontal activity further strengthens this link. These results, observed during BAT, provide a basis for investigating the central-peripheral fatigue interplay and its implications for stroke recovery.

Our finding of a positive correlation between left biceps activity regularity and right motor cortex activation, with similar trends in the left motor cortex, supports the concept of lateralized cortical reorganization after stroke. While low entropy in the unaffected limb suggests more precise control (Jin et al., 2024), our focus on BAT differentiates our work from studies on unilateral movement (Wang et al., 2023), potentially explaining discrepancies with respect to reports on lower limb cycling (Jin et al., 2024) and cortico-muscular coupling during unilateral upper limb movements (Xi et al., 2021). Our observation of increased contralateral motor region activation, and a trend towards increased ipsilateral activation, aligns with the intracerebral disequilibrium theory (Murase et al., 2004). Furthermore, the negative correlation between left bicep regularity and right occipital activation reflects the role of the occipital lobe in visuomotor integration during BAT. Finally, the positive correlation between right upper limb entropy and bilateral motor region activation reflects varied cortical reorganization patterns in bilateral hemiplegia (Muller et al., 2024). These findings highlight the complex motor-visual interplay in bilateral stroke tasks.

### Limitations

The majority of the stroke patients were men, with a significant proportion of women in the control group. The notable gender disparity and limited sample size led to substantial heterogeneity between the groups, thus warranting a cautious interpretation of the study results. Additionally, we only conducted a linear correlation analysis of the electromyography and cerebral oxygenation signals during the upper limb coordination tasks, and plan to explore the more complex nonlinear relationship between the two variables in future research. While the current investigation had a cross-sectional design, future longitudinal studies and follow-up work are warranted to gain a more profound understanding of the mechanisms underlying the effects of BAT.

Subgroup analysis for cerebral hemorrhage and ischemia was not performed because of the small sample size, which may have led to underpowered results. Despite differences in their pathophysiological mechanisms, these conditions often result in similar clinical outcomes, such as unilateral upper limb hemiparesis. In clinical practice, rehabilitation strategies for bilateral upper limb training are generally the same for ischemic and hemorrhagic stroke patients. Therefore, this study focused on the overall effect of the intervention in all stroke patients as a single group. Larger cohort studies are needed to explore potential differences between these stroke types.

We found that stroke patients exhibited asymmetric cerebral oxygen responses compared with healthy individuals, such that those with more severe upper limb motor dysfunction showed lower and flatter cerebral oxygen activation. Furthermore, we identified certain linear correlations between cerebral oxygenation and electromyography signals in stroke patients. These findings provide a foundation for future research. They may guide the development of personalized limb coordination training programs for stroke patients based on cerebral oxygen and electromyography signal feedback.

### Conclusion

Our findings reveal a significant correlation between neural and muscular activities during BAT. Additionally, we observed lateralized cerebral oxygenation activation in stroke patients during bilateral upper limb exercises, with a marked reduction in functional connectivity compared with the findings in healthy individuals. Furthermore, in stroke patients, we found a notable decrease in integrated electromyography, RMS, and MPF values of the upper limbs, accompanied by a significant increase in FE within the flexor muscles on the paretic side. These insights contribute to a deeper understanding of the central and peripheral mechanisms underlying bilateral upper limb movements.

**Additional file:** *Open peer review report 1.*

OPEN PEER REVIEW REPORT 1

## Data Availability

*The data are available from the corresponding author on reasonable request*.
