# Supplementary material for: Investigating the correlation between neural and muscular activities during bilateral arm training in stroke survivors: A cross-sectional study
Source: Neural Regen Res. 2025 Jun 19;21(7):3026–34. doi: 10.4103/NRR.NRR-D-24-01279 (PMC13378940; doi:10.4103/NRR.NRR-D-24-01279)
Supplement: Supplementary file 1 [file NRR-21-3026_Suppl1.pdf]

OPEN PEER REVIEW REPORT 1

Name of journal: Neural Regeneration Research
Manuscript NO: NRR-D-24-01279
Title: Investigating the correlation between neural and muscular activities during bilateral arm training in stroke survivors
Reviewer's Name: Jonathan M. Borkum
Reviewer's country: USA

COMMENTS TO AUTHORS

Page 5, Table 1. "Infraction" should be "Infarction"

Page 12, Lines 31-37: "... a higher IEMG and RMS value represented the recruitment of more motor units and better muscle strength." Also, higher firing rates within a motor unit?

Page 16. "FDR-corrected" Does this refer to the Bonferroni correction? If so, please indicate.

Page 17, Line 9: "... within channel 24 and 25..." I think it will make it easier for the reader to repeat there that these refer to the right and left motor areas, respectively

Table 4. If I am understanding correctly, there is no correction for the fact that 480 correlations were calculated. That's okay for an exploratory study but I feel it should be mentioned in the discussion.

Page 27, Line 1: "...increased MPF..." Wasn't the MPF decreased in the stroke patients?
